# Comparing the effectiveness of different DNA extraction methods in MX‐80 bentonite

**DOI:** 10.1111/1758-2229.70047

**Published:** 2024-11-24

**Authors:** Kristel Mijnendonckx, Carla Smolders, Deepa Bartak, Trung Le Duc, Mar Morales‐Hidalgo, Cristina Povedano‐Priego, Fadwa Jroundi, Mohamed L. Merroun, Natalie Leys, Katerina Cerna

**Affiliations:** ^1^ Unit of Microbiology Belgian Nuclear Research Centre Mol Belgium; ^2^ Department of Applied Biology, Institute for Nanomaterials, Advanced Technologies and Innovation Technical University of Liberec (TUL) Liberec Czech Republic; ^3^ Department of Microbiology University of Granada Granada Spain

## Abstract

Approaches to DNA extraction play a crucial role in determining the variability of results obtained through 16S rRNA amplicon sequencing. Particularly, clay‐rich samples can impede the efficiency of various standard cultivation‐independent techniques. We conducted an inter‐laboratory comparison study to thoroughly assess the efficacy of two published DNA extraction methods (kit‐based and phenol‐chloroform‐based) specifically designed for bentonite samples. To this end, we spiked Wyoming MX 80 bentonite with two different mock communities and compared the obtained DNA yield and purity, the presence of contaminants and the community profile. Our findings suggest that both methods are equally viable, with the best choice depending on the specific requirements of the downstream analysis. However, it is crucial to maintain consistency in the chosen method, as comparing results becomes challenging, particularly in the presence of bentonite. In summary, our study emphasizes the significance of standardized DNA extraction methods and underscores the importance of validating these methods using appropriate controls when studying microbial communities with 16S rRNA amplicon sequencing, particularly in environments characterized by low biomass and clay‐rich compositions. Additionally, slight modifications to one of the extraction methods can substantially enhance its efficiency.

## Graphical abstract

We conducted an inter‐laboratory comparison of two slightly modified DNA extraction methods on sterilized Wyoming MX‐80 bentonite, amended with two defined mock communities. The choice between the two methods is not critical, as each has its advantages and limitations. Additionally, our findings highlight the importance of including appropriate controls when working with challenging low‐biomass samples.

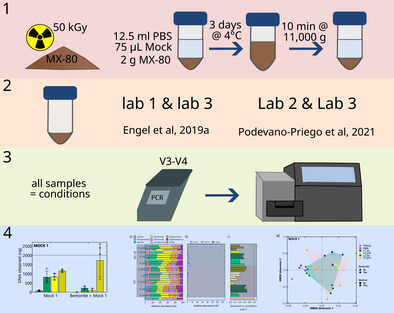

## INTRODUCTION

With the recent advances in high‐throughput ‘omics’ techniques, sequencing technologies have emerged as key tools for studying microbial communities. To date, 16S rRNA amplicon sequencing is by far the most widely used approach to investigate microbial composition dynamics (Starke et al., 2021). It has provided valuable insights into microbial diversity across various environments (Ainsworth et al., 2015; Bachran et al., 2019; Lopez‐Fernandez et al., 2015; Van Eesbeeck et al., 2021). However, it is well‐known that the outcome of 16S rRNA amplicon sequencing can be affected by the DNA extraction method, primer design, PCR amplification, sequencing artefacts and bioinformatics analysis (Costea et al., 2017; Karst et al., 2018; Kebschull & Zador, 2015; Zielinska et al., 2017). Consequently, comparing results obtained using different methods is not always straightforward (Abellan‐Schneyder et al., 2021). Additionally, special care must be taken when dealing with low‐biomass samples, because they are highly susceptible to contamination during sample preparation, DNA extraction and subsequent manipulations (Salter et al., 2014).

One example of such a challenging low‐biomass environment is bentonite clay, which is considered as the backfill material in engineered barriers for the geological disposal of nuclear waste in many countries (Sellin & Leupin, 2013). Depending on the conditions, bentonite might contain 10^2^–10^6^ CFU/g in total and ~10^6^ viable cells/g (Burzan et al., 2022; Engel, Ford, et al., 2019; Stroes‐Gascoyne et al., 2011; Vachon et al., 2021). This is several orders of magnitude lower than, for example, soils, which can harbour up to 10^10^ bacterial cells/g (Raynaud & Nunan, 2014). The highly compacted bentonite buffer in a geological waste repository is expected to limit microbial activity due to its high swelling pressure and low water activity (Pedersen et al., 2000; Stroes‐Gascoyne et al., 2010). However, several in situ experiments demonstrated the persistence of microorganisms within bentonite (Burzan et al., 2022; Chi Fru & Athar, 2008; Engel, Ford, et al., 2019). Moreover, a significant number of bacterial cells are expected to remain viable under the harsh conditions anticipated after repository closure, which include increased pressure, heat and irradiation (Haynes et al., 2018). Microbially‐influenced corrosion by sulfate‐reducing bacteria (SRB) is one of the primary microbial processes of concern regarding the geological disposal of nuclear waste (King et al., 2021). To gain a comprehensive understanding of the impact of microbial processes during geological disposal of nuclear waste, recent studies have employed a combination of cultivation‐dependent and cultivation‐independent approaches (Bartak et al., 2023; Beaver et al., 2022; Burzan et al., 2022; Vachon et al., 2021). However, an additional challenge of working with clay‐rich samples is that they can hamper the efficiency of several standard cultivation‐independent methods. Clay particles are known to tightly adsorb organic and inorganic phosphorous compounds (Cai et al., 2007). Since the DNA backbone is rich in phosphate, DNA molecules tend to adhere to clay adsorption sites. This also includes DNA released after cell lysis which can be adsorbed on clay particle surfaces before the DNA extraction procedure is finalized. Consequently, this significantly hinders the efficiency of DNA extraction (Frostegård et al., 1999). On the other hand, the high adsorption capacity enables clay particles to preserve DNA molecules over long time periods (Frostegård et al., 1999; Romanowski et al., 1991). An additional difficulty with DNA extractions from bentonite is its swelling upon addition of lysis buffer, hindering the complete release of microbial cells from the bentonite matrix (Povedano‐Priego et al., 2021). A possible solution is indirect DNA extraction methods where intact cells are recovered from the sample before lysis and DNA extraction (Högfors‐Rönnholm et al., 2018). However, these methods introduce bias because separation treatments exhibit varying efficiencies depending on the type of microorganisms involved (Holmsgaard et al., 2011), albeit rather limited in some studies (Courtois et al., 2001; Delmont et al., 2011). Nevertheless, direct DNA extraction methods are preferred and are used most frequently. To overcome the limitations of direct methods from clay‐rich samples, blocking agents such as skim milk are often added. However, their usage may inadvertently introduce varying concentrations of contaminating DNA (Ikeda et al., 2008; Takada‐Hoshino & Matsumoto, 2004).

To date, a few DNA extraction methods have been published for clay; however, they have either been validated with spiking DNA of a single strain (Engel, Coyotzi, et al., 2019), cells of only two strains (Stone et al., 2016) or not validated at all (Chi Fru & Athar, 2008; Lopez‐Fernandez et al., 2015; Povedano‐Priego et al., 2021). Validation using a mock community is essential, as applying different DNA extraction methods to clay samples has resulted in significant variation in the outcomes of 16S rRNA amplicon sequencing (Mijnendonckx et al., 2021). Therefore, a more standardized research methodology is needed to study microbial communities in low biomass, clay‐rich environments. Two distinct DNA extraction methods (Engel, Coyotzi, et al., 2019; Povedano‐Priego et al., 2021) have shown suitability for clay samples (Mijnendonckx et al., 2021). The method described by Engel, Coyotzi, et al. (2019) is a kit‐based approach involving a combination of sodium dodecyl sulfate (SDS)‐based and mechanical lysis, followed by DNA binding and washing on a silica column. On the other hand, the method proposed by Povedano‐Priego et al. (2021) includes pre‐treatment with phosphate buffer and glass beads, chemical and enzymatic lysis using a cocktail containing polyvinylpyrrolidone (PVP), SDS, proteinase K, lysozyme and mechanical/thermal shocks, followed by DNA precipitation with agents including phenol and chloroform. Both protocols offer an optional additional step for DNA concentration. Validation of the two methods was previously only performed with one replicate of Opalinus Clay spiked with a mock community (Mijnendonckx et al., 2021). Here, we performed a detailed inter‐laboratory comparison of these two DNA extraction methods with slight laboratory‐specific modifications on sterilized Wyoming MX‐80 bentonite amended with two defined mock communities. Our objective was to compare the performance of both DNA extraction approaches by means of evaluating DNA yields, amplifiability of obtained DNA, the community profile and the presence of contaminants in the context of the clay and the methods considered.

## EXPERIMENTAL PROCEDURES

### Mock communities

**FIGURE 1:**
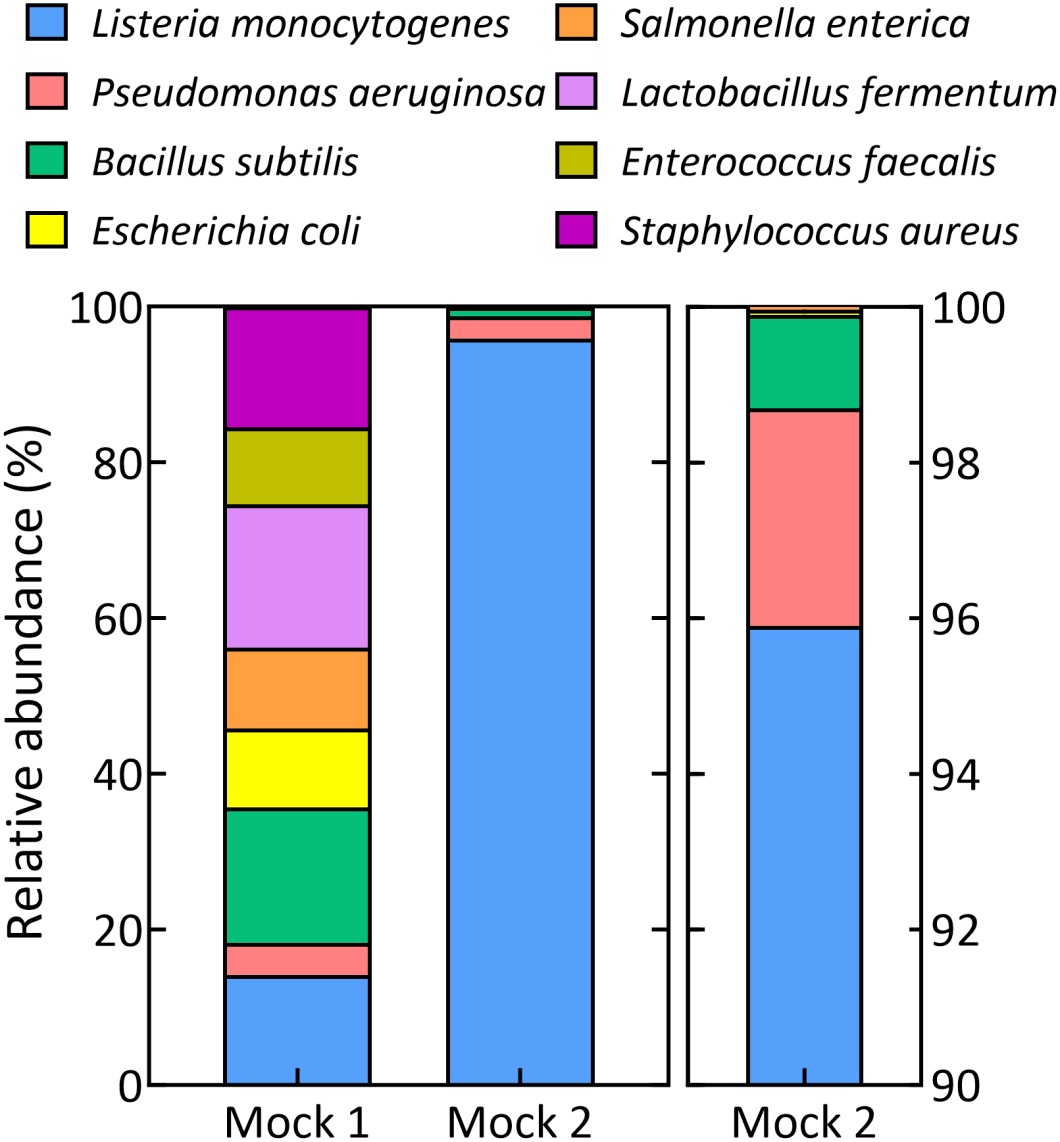
Overview of the theoretical composition of both mock communities used in this study.

We selected two commercial microbial community standards consisting of DNA or intact cells of three Gram‐negative bacteria, five Gram‐positive bacteria and two yeast species with varying size and cell wall recalcitrance (Table S1; ZymoBIOMICS Mock Community standards, Zymo Research Corporation, Irvine, USA). Mock 1 (D6300) contains a total cell concentration of ca. 1.4 × 10^10^ cells/mL and a linear distribution of each of the species, whereas Mock 2 (D6310) contains ca. 1.5 × 10^9^ cells/mL with a logarithmic distribution of the different strains (Figure 1).

### Bentonite

Wyoming MX‐80 bentonite was provided by the National Cooperative for the Disposal of Radioactive Waste (NAGRA), sterilized in the same facility, and used by all three participating labs. Sterility was achieved by gamma irradiation with a total dose of 50 kGy. Sterility validation was performed by cultivation in each lab. To this end, 5 mL PBS was supplemented to 0.5 g MX‐80 and stirred for 30 min. Afterwards, 100 μL of a 1/10 dilution series was cultivated in liquid and solid R2A medium (Reasoner & Geldreich, 1985) and incubated at 30°C in aerobic for 3days and anaerobic conditions for 3 weeks. In addition, the presence of SRB was probed by cultivation in modified Postgate's B medium (Schwartz, 1985) and incubation at 30°C under anoxic conditions for 28 days.

### Experimental setup

Two grams of sterile MX‐80 bentonite was supplemented with 12.5 mL Phosphate‐buffered saline (PBS), and either 75 μL of Mock 1 or Mock 2 or none (control). All setups were performed in triplicate. Samples were thoroughly mixed by vortexing and incubated for 3 days at 4°C to enable water absorption into and cell interaction with bentonite. After the incubation step, samples were centrifuged at 11,000 rpm for 10 min. The supernatant was discarded and the pellet was used for following DNA extraction methods. To elucidate the possible bias introduced by the presence of bentonite on DNA recovery, extractions were also performed on 75 μL of both mock communities.

### DNA extraction methods

Three independent laboratories used distinct DNA extraction methods to have multiple independent replicates for validation of the two methods. Lab 1 used the kit‐based approach (Engel et al., 2019), Lab 2, the method based on phenol‐chloroform extraction (Povedano‐Priego et al., 2021) and Lab 3 applied both extraction methods. However, there were slight modifications to both original protocols which are described below.

#### Lab 1⸺Kit‐based

Lab 1 used the protocol by Engel, Coyotzi, et al. (2019) based on the DNeasy® PowerMax® Soil Kit (Qiagen, Germany) with minor modifications. Briefly, 15 mL PowerBead solution was added to each bentonite pellet and the sample was vortexed for 1 min. After addition of 1.2 mL lysis solution, samples were vortexed vigorously for 30 s followed by vortexing in a horizontal vortex adapter at maximum speed for 10 min. Subsequently, the samples were placed in a shaking water bath at 65°C for 30 min. Afterwards, the manufacturer's protocol was followed and the DNA was eluted into 1 mL elution buffer (10 mM Tris). The extracted DNA was further purified and concentrated using the Genomic DNA Clean & Concentrator™ Kit (Zymo Research, USA) following the manufacturer's protocol to obtain 50 μL as the final volume. The extracted DNA was subsequently quantified using a Qubit 2.0 fluorometer (Invitrogen, Life Technologies, USA) according to the manufacturer's protocol.

#### Lab 2⸺Phenol‐chloroform

Lab 2 performed the extractions following the optimized protocol for total DNA isolation from bentonite as previously described by Povedano‐Priego et al. (2021). The bentonite pellet obtained after centrifugation was distributed in portions of 0.3 g in individual 2‐mL screw‐cap micro‐centrifuge tubes. This protocol consists of a pre‐treatment using 400 μL Na_2_HPO_4_ (0.12 M, pH 8.0) followed by chemical and enzymatic lysis by the addition of 600 μL of lysis buffer (100 mM Tris–HCl [pH 8.0], 100 mM EDTA [pH 8.0], 100 mM NaCl, 1% PVP and 2% SDS), 24 μL freshly made lysozyme (10 mg/mL) and 2.5 μL proteinase K (20 mg/mL) to each tube. Mechanical lysis was performed twice using a FastPrep® FP120 (MP Biomedicals) bead‐beater at 5.5 m s^−1^ for 45 s. Afterwards, samples were incubated at 37°C for 30 min first and then at 60°C for 1 h. Then, samples were centrifuged at 14,000 g for 5 min and all supernatants for the same sample were pooled in a 15 mL tube. An additional mechanical lysis step was performed with the bentonite pellet using 1 mL lysis buffer, followed by another centrifugation step. One volume of phenol:chloroform:isoamyl alcohol (25:24:1 v/v) was added to the tubes and centrifuged at 1500 g for 10 min at 4°C. This step was followed by a modification of the protocol described by Povedano‐Priego et al. (2021): the supernatants were transferred to a new tube and one volume of chloroform was added and mixed. Tubes were again centrifuged. Afterwards, the next steps were performed following the extraction method in Povedano‐Priego et al. (2021). Total DNA was resuspended in 35 μL milli‐Q water and quantified on a Qubit 3.0 Fluorometer (Life Technologies) and stored at −20°C until further processing.

#### Lab 3⸺Kit‐based

Lab 3 followed the protocol of Lab 1 with some minor modifications. After incubation in a water bath at 65°C for 30 min, samples were homogenized by vortexing the tubes 10 min at maximum speed with a Vortex adapter cat 13000‐V1 (Qiagen, the Netherlands). Then, we followed the same protocol until the elution step. Purified DNA was eluted in 2.3 mL of the provided elution buffer (10 mM Tris). Nucleic acids were precipitated using 4 μL/mL Genelute‐LPA (25 mg/mL; Sigma‐Aldrich, Belgium), 0.1 volumes of 5 M NaCl, 1 volume of isopropanol, gently mixed by inverting the tubes and stored at −20°C overnight. Precipitated DNA was pelleted by centrifugation at 13,000 g for 30 min at 4°C and then washed with 80% ice‐cold ethanol (stored at −20°C). Finally, pellets were air‐dried in a laminar flow for 15 min and finally suspended in 125 μL of elution buffer (10 mM Tris). DNA concentration was measured with the Quantifluor dsDNA sample kit (Promega, the Netherlands).

#### Lab 3⸺Phenol‐chloroform

The procedure of Povedano‐Priego et al. (2021) was followed with some modifications. Mechanical lysis was performed using a TissueLyser II (Qiagen, Belgium) for 10 min at 30 Hz. In addition, after the extraction with phenol:chloroform:isoamyl alcohol (25:24:1 v/v), the upper (aqueous) phase was transferred to a new tube and washed by adding one volume of chloroform:isoamyl alcohol (1:1 v/v). Tubes were again centrifuged at 1,500 g for 10 min at 4°C and the supernatants were transferred to a new tube. Afterwards, DNA was precipitated by adding 1 volume of 75% isopropanol and 1/10 volume of 3 M sodium acetate (pH 5.3) and overnight incubation at −20°C. Afterwards, the sample was centrifuged 30 min at 5,000 g at 4°C, the pellet was washed with 5 mL of an 80% ice‐cold ethanol solution (stored at −20°C) and centrifuged for 5 min at 10,000 g. The supernatant was discarded and the pellet was dried overnight at 30°C. Finally, all DNA pellets obtained for one replicate were pooled and dissolved in 500 μL milli‐Q water. Subsequently, the sample was applied on a 100 kDa Amicon filter unit (Merck, Belgium) and centrifuged for 10 min at 14,000 g. The pellet was washed twice with 500 μL milli‐Q water. Finally, the pellet was eluted by centrifugation for 2 min at 1,500 g. DNA concentration was measured with the Quantifluor dsDNA sample kit (Promega, the Netherlands).

### 16S rRNA amplicon sequencing

All DNA samples were sent to Lab 3 where all the PCRs were performed to minimize variability that could be introduced by that step. The V3‐V4 region of the 16S rRNA gene was amplified with primers 341F (5′‐CCTACGGGNGGCWGCAG‐3′) and 785R (5′‐GGACTACHVGGGTATCTAATCC‐3′) (Klindworth et al., 2013) using the Phusion High‐Fidelity Polymerase (Thermofisher Scientific, Belgium). Primers contained an Illumina adapter overhang sequence: 5′‐TCGTCGGCAGCGTCAGATGTGTATAAGAGACAG‐3′ for the forward primer and 5′‐GTCTCGTGGGCTCGGAGATGTGTATAAGAGACAG‐3′ for the reverse primer. PCR conditions were as follows: 1 min at 98°C followed by 30 cycles of 10 s at 98°C, 30 s at 62°C and 1 min at 72°C followed by a final extension of 10 min at 72°C. Five nanogram was used as DNA template in all samples except when the concentration was too low, where 5 μL was used. Initially, 70 samples were used for PCR amplification, including 48 samples spiked with a Mock community, 12 sterile bentonite samples, two negative kit controls, two no‐template PCR controls (NTC) and three replicates of each Mock community standard consisting of DNA instead of intact cells, processed according to the manufacturer's recommendations (Table S1; ZymoBIOMICS Mock Community standards D6306 and D6311, Zymo Research Corporation, Irvine, USA) to examine possible PCR bias. PCR results were evaluated via gel electrophoresis by loading 5 μL (at least 250 ng) of each sample onto a 1% agarose gel. PCR products from samples that were positive were purified with the Wizard® SV Gel and PCR Clean‐Up System (Promega, The Netherlands) according to the manufacturer's protocol. Samples with a DNA yield above the detection limit but with a negative result after PCR amplification were further purified by either heating to 50°C for 1 h, diluting 20, 40 or 80 times, drop dialysis or on a 100‐kDa Amicon® Ultra filter device (Merck, Belgium). For drop dialysis, a standard Petri dish was half‐filled with milli‐Q water. A nitrocellulose membrane (pore size 0.025 μm, diameter 25 mm, Merck, Belgium) was floated on the water. The aliquot of the DNA sample was pipetted on the membrane and left to dialyze for 1 h. Afterwards, the sample was recovered from the top of the membrane. Purification on a 100‐kDa Amicon® Ultra filter device (Merck, Belgium) was performed by applying the sample on the column and centrifuging it for 10 min at 14,000 g. The sample was washed twice with 500 μL milli‐Q water. To recover the concentrated sample, the Amicon® filter was placed upside down in a clean microcentrifuge tube and centrifuged for 2 min at 1,500 g.

All samples were sequenced on the Illumina MiSeq platform according to the manufacturer guidelines at BaseClear B.V (the Netherlands).

### Bioinformatics and statistical analyses

Primers were first removed from the 16S rRNA gene amplicon sequencing data using cutadapt (Martin, 2011). Subsequently, raw reads were processed according to the DADA2 pipeline with recommended settings (Callahan et al., 2016). Briefly, reads with ambiguous, poor‐quality bases and more than two expected errors were discarded. The paired reads were merged, chimeras were identified and removed. Only amplicon sequence variants (ASV) with more than two reads were retained. Taxonomy was assigned to the ASVs using the naive Bayesian classifier method implemented in DADA2 with the Silva taxonomic training dataset (version 132) as a reference (Callahan, 2018).

Potential contaminant ASVs were identified through the Decontam (v.1.6.0) R package (Davis et al., 2018). Specifically, the ‘combined’ method was used, where frequency and prevalence probabilities are combined with Fisher's method and used to identify contaminants. For samples with a DNA concentration below the detection limit but a positive sequencing result, the detection limit of the DNA measurement method was used to calculate the total amount of DNA in the sample. With a probability threshold of 0.5, we identified 10 contaminant ASVs and excluded them from subsequent analyses.

The 16S rRNA amplicon sequencing data were further analysed in R version 4.3.0 with the R package phyloseq (McMurdie & Holmes, 2013). Subsampling was performed based on the lowest number of reads obtained over the different samples amended with a Mock community, that is, a coverage of 8403 reads. Rarefaction curves indicate that this level of subsampling adequately represented the bacterial diversity in the samples (Figure S1). The package chkMocks was used to compare the composition obtained in each condition with the theoretically expected composition (Sudarshan et al., 2023). The β‐diversity was calculated by non‐metric multidimensional scaling (NMDS) with Bray‐Curtis distances with the command ‘ordinate’. Afterwards, a distance matrix of these data was calculated with the command ‘distance’. This distance matrix was used to perform a permutation test for homogeneity of multivariate dispersions using the command ‘betadisper’ in the package vegan (Oksanen et al., 2020). Permutational multivariate analysis of variance (PERMANOVA) using the ‘adonis’ at 999 permutations and *α* = 0.05 were performed to test whether there was a difference between the DNA extraction methods and if the bentonite had an effect on the outcome. Pairwise multilevel comparison on microbial community structure between samples was performed with the R package pairwiseAdonis with FDR (False Discovery Rate) correction to the *p*‐values. The datasets generated and analysed during this study are available in the NCBI Sequence Read Archive (SRA) repository (PRJNA1054184).

## RESULTS

### DNA yield

**FIGURE 2:**
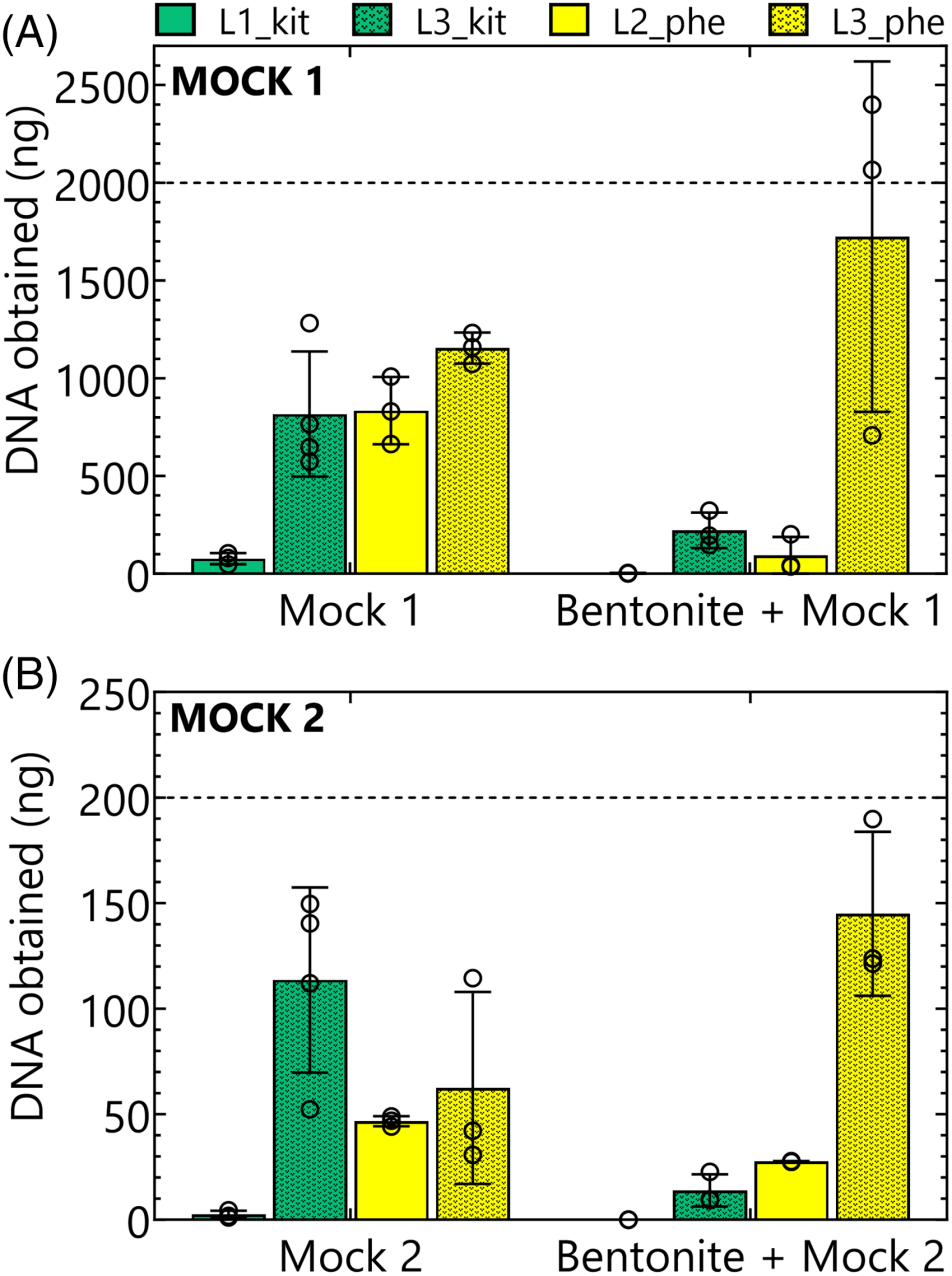
Total amount of DNA obtained for samples with (A) Mock 1 or bentonite spiked with Mock 1 and (B) Mock 2 or bentonite spiked with Mock 2. The theoretical DNA yield expected in all samples following the procedure recommended by the manufacturer is given by a dotted line. Samples represent the average of three replicates except for L3_kit Mock 1 and L3_kit Mock 2 samples, which include four replicates. Kit‐based methods are shown in green, while phenol‐chloroform‐based methods are shown in yellow. Individual results of the replicates are indicated with open circles.

The theoretically expected total DNA yield should have been ca. 2 μg and 200 ng of DNA for all samples amended with Mock 1 and Mock 2, respectively, if the DNA extraction protocol recommended by ZymoResearch (i.e., ZymoBIOMICS™ DNA Miniprep [D4300]) was applied. Each laboratory successfully obtained quantifiable DNA concentrations from all samples spiked with a Mock community, except for Lab 1 when analysing bentonite samples spiked with Mock 2. However, the results show that even in the absence of bentonite, DNA yields were lower than half of the expected amount. In the presence of bentonite, this difference further increased, except for DNA extracted by the phenol‐chloroform method from lab 3 where bentonite did not adversely affect the efficiency of DNA extraction. However, a large variation among the replicates was observed (Figure 2). DNA yields from kit‐based methods employed by two labs differed with and without bentonite. Lab 3 obtained 10 times more DNA from Mock 1, and in the presence of bentonite and also for all samples spiked with Mock 2, an 80‐fold difference was observed. Minor differences were observed when using the phenol‐chloroform‐based method for Mock‐only sample extractions, but similarly high differences were observed in spiked bentonite samples where lab 3 achieved a notably higher total DNA yield than lab 2 (ca. 18 and 5 times more for samples spiked with Mock 1 and Mock 2, respectively). It is worth mentioning that the kit‐based extraction method failed to yield any measurable amount of DNA from the sterile unspiked bentonite samples. Conversely, the phenol‐chloroform‐based method successfully extracted DNA from five of the six replicates of sterile unspiked samples (Figure S2).

**FIGURE 3:**
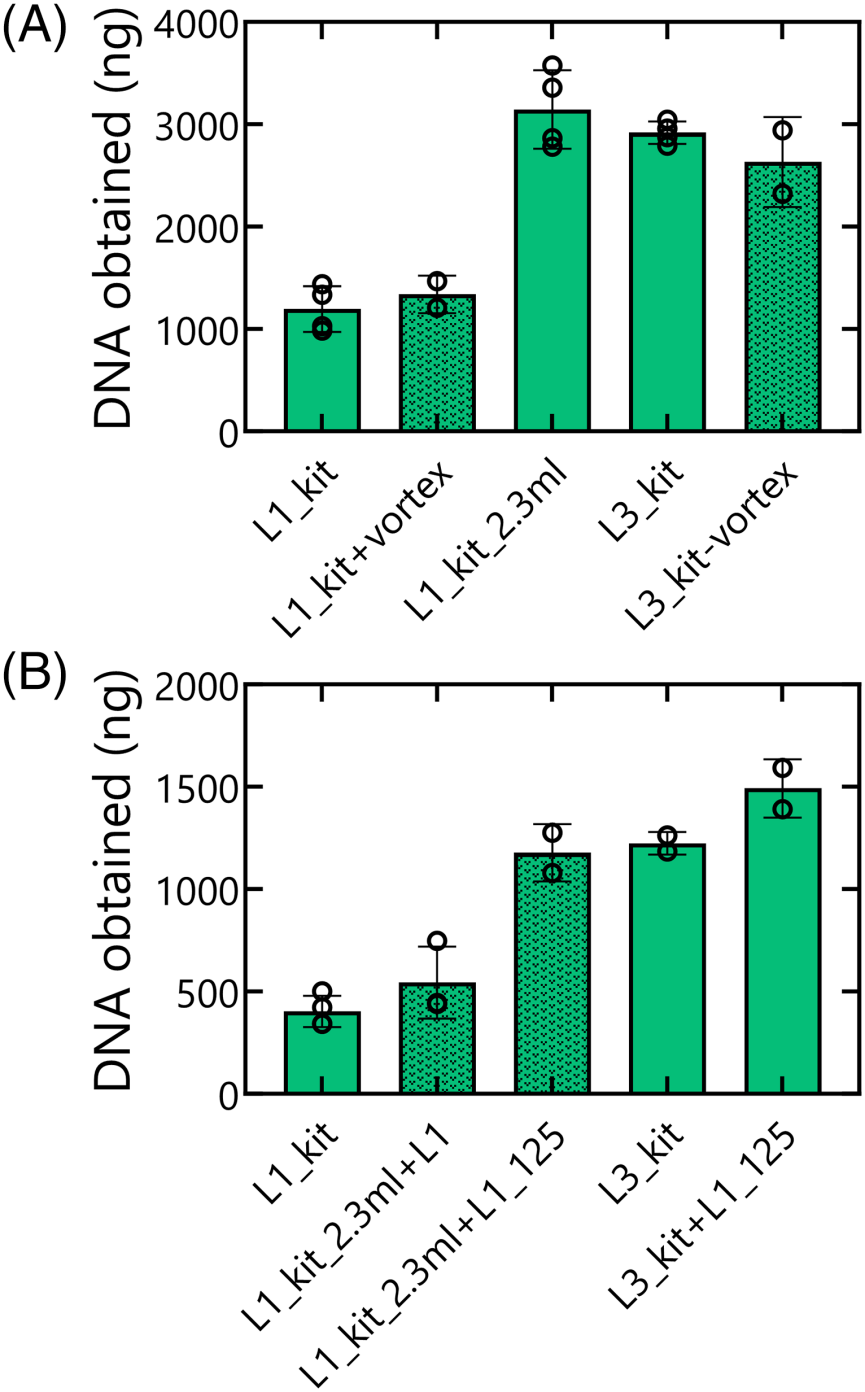
Total amount of DNA obtained for Mock 1 samples processed with kit‐based approaches after (A) intermediate elution; (B) final elution. Results of the lab 1 procedure are indicated with full bars, lab 3 is indicated with dotted bars. The differences with the original protocols are mentioned after the L1_kit or L3_kit. Individual results of the replicates are indicated with open circles.

The large difference between the kit‐based methods particularly in the absence of bentonite may result from the following four factors. First, lab 3 applied a vortexing step after incubation in the water bath. Second, lab 3 used a larger volume of elution buffer (2.3 mL) than lab 1 (1 mL). Third, lab 3 used precipitation methods with LPA for DNA concentration, whereas lab 1 used the Genomic DNA Clean & Concentrator™ Kit. Finally, the volume used for the final elution was 50 μL in case of lab 1 and 125 μL in case of lab 3. To elucidate the effect of each of these differences, lab 3 conducted different combinations of kit‐based extraction protocols on Mock 1 without bentonite. In first instance, we investigated the effect of the additional vortex step of lab 3 and the increased volume (2.3 mL instead of 1 mL) at the intermediate elution. The total amount of DNA was measured after the intermediate elution before the additional cleaning of the DNA. Including the vortexing step in the procedure of lab 1 or removing it from the protocol of lab 3 did not increase DNA yield. However, eluting in 2.3 mL instead of 1 mL increased the amount of DNA matching results observed with the procedure of lab 3 (Figure 3). Next, the protocol of lab 1 was performed with an intermediate elution in 2.3 instead of 1 mL and a final elution in 50 and 125 μL. This indicated that upscaling of the volumes seems to be the most explicatory factor that explains the difference in DNA yield between lab 1 and lab 3. Finally, as using a complete kit‐based approach is less time‐consuming and is expected to be more standardized and reproducible, we combined the kit‐based protocol of lab 3 with the Genomic DNA Clean & Concentrator™ Kit (Zymo Research, USA) of lab 1 and eluted in 125 μL. This also resulted in similar yields (Figure 3). It is noteworthy that when Lab 1's procedure was replicated in Lab 3 even by the same individual, the DNA yield was five times higher than when conducted in Lab 1, highlighting the potential impact of differences in lab equipment (Figures 2 and 3).

### PCR

Initially, up to 62 DNA samples were applied for PCR amplification, but the samples used for the protocol optimizations described in the previous paragraph were not included. PCR was successful for all 15 samples originating from lab 1 (kit‐based method), including both negative kit control samples and the sterile unspiked bentonite samples (Table S2). Similarly, 16 of the 17 samples from lab 3 using the kit‐based approach were positive, including two unspiked bentonite samples. Only three of the 15 samples from lab 2 using the phenol‐chloroform approach were positive, namely the three replicates of Mock 1 without bentonite. Finally, nine of the 15 samples of lab 3 processed with the phenol‐chloroform method were positive (Table S2). In addition to the 62 samples, we included two NTC in the PCR reaction. It is important to note that these NTC samples also yielded a band. Finally, we included PCR reactions on three replicates of each ZymoBIOMICS Microbial Community DNA Standard (Zymo Research Corporation, Irvine, USA).

Samples with a DNA yield above the detection limit but with a negative result after PCR amplification underwent additional purifications. The three replicates of bentonite spiked with Mock 2 extracted with the phenol‐chloroform‐based method by lab 3 underwent additional purification through drop dialysis, followed by diluting the purified samples 40 times. This resulted in positive PCR amplification for two out of three replicates after a 40‐times dilution. As the samples of lab 2 visually differed from the other samples (Figure S3), attempts were made to improve the PCR results by first heating the samples to 50°C and diluting them 20, 40 or 80 times. However, these measures did not yield a positive PCR reaction. Therefore, samples were purified by other methods. At first, since the phenol‐chloroform extraction method differed between labs 2 and 3 from the phenol‐chloroform step onwards, the DNA extraction from the pellet was repeated starting from there. This resulted in a successful amplification of three samples: one replicate of bentonite spiked with Mock 1, one replicate of Mock 2 and one replicate of the sterile bentonite samples. Subsequently, an additional purification step was performed using drop dialysis, enabling the amplification of the remaining two replicates of Mock 2. The other seven samples of lab 2 remained negative (Table S2).

As all these purification steps could affect the outcome of further analysis, we included additional controls where possible. To this end, Mock 1 samples of lab 2 that were already positive after the first PCR, were also purified starting from the phenol‐chloroform step onwards and again amplified to compare both results. In addition, to check the effect of the dilution, the two replicates of Mock 2 processed by lab 2 that were positive after drop dialysis, were also diluted 40 times, amplified again and the result was compared to the undiluted sample.

In summary, successful PCR was achieved for a total of 41 out of 48 samples spiked with a mock community, 6 of the 12 sterile bentonite samples and the 2 negative kit controls. An overview of all samples, DNA yields and the measures needed to obtain a positive PCR reaction are given in Table S2. All these samples, together with the PCR reactions performed on three replicates of each ZymoBIOMICS Microbial Community DNA Standard (Zymo Research Corporation, Irvine, USA), 2 NTC PCR controls, controls to assess the impact of the additional phenol‐chloroform extraction, drop dialysis and dilution, totalling 66 samples, were sent for 16S rRNA amplicon sequencing.

### 16S rRNA amplicon sequencing

**FIGURE 4:**
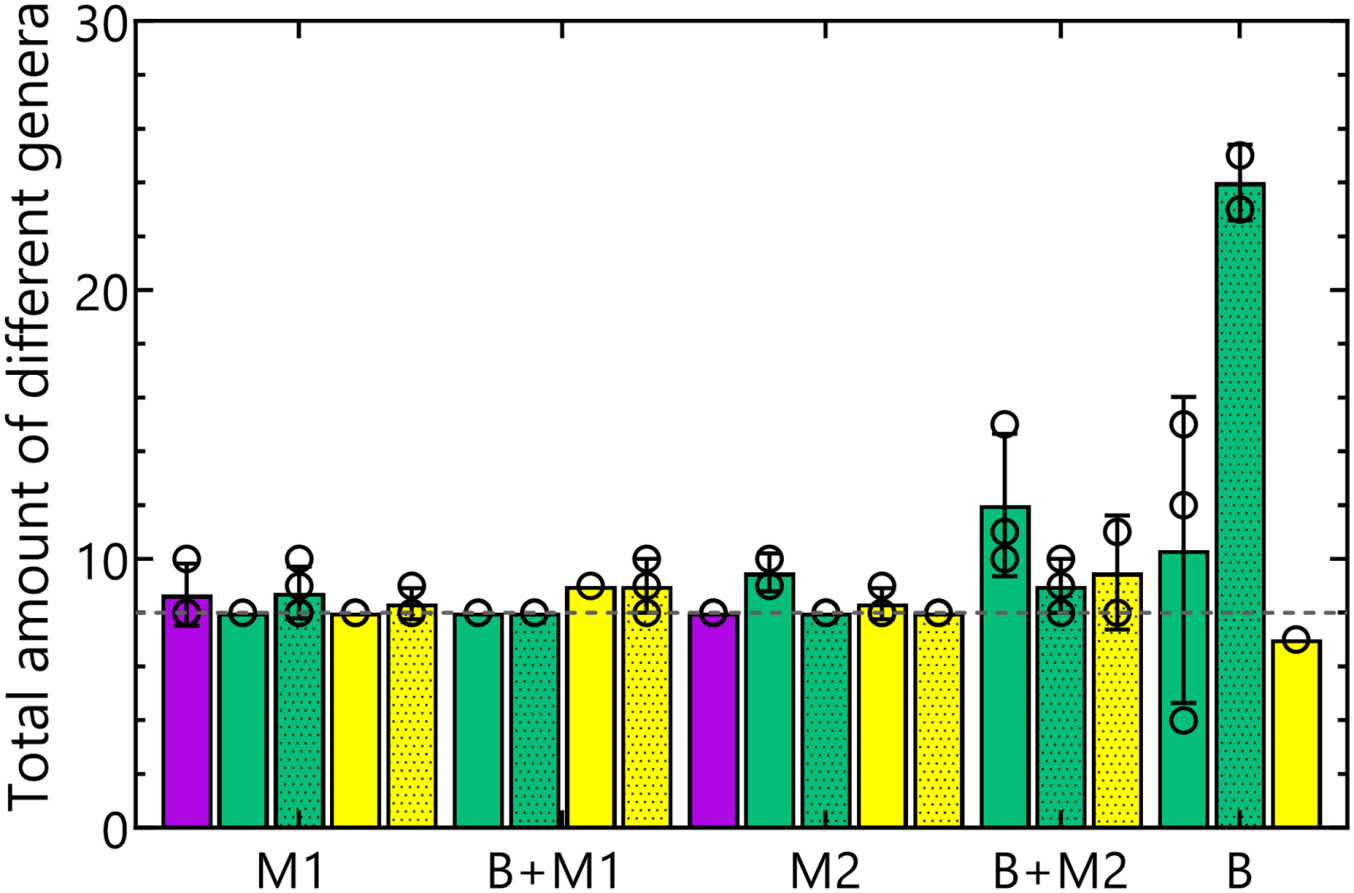
Number of genera identified in the samples spiked with Mock 1 in the absence of bentonite (M1) or in the presence of bentonite (B + M1); in the samples spiked with Mock 2 in the absence of bentonite (M2) or in the presence of bentonite (B + M2); in unamended sterile bentonite (B). A dotted line indicates the expected number of genera present in the mock samples. Results of the DNA Mock are shown in purple; kit‐based methods are shown in green, while phenol‐chloroform‐based methods are shown in yellow. Individual results of the replicates are indicated with open circles.

In both NTC (lab 3) and the two kit controls (lab 1), *Cupriavidus* was the predominant genus, constituting over 99% of the total relative abundance (Supplementary Figure 4). In fact, 10 ASVs were identified as true contaminants and were removed from the dataset in all subsequent analyses (Table S3). After eliminating the contaminants, a total of 123 ASVs comprising 59 genera were identified. In most of the samples spiked with a mock community, only the eight genera present in the Mock were identified (Figure 4). However, there is a discrepancy between the number of identified ASVs and the expected number (Figure S5), which could be mainly attributed to the fact that several genera were defined by multiple ASVs. Nevertheless, the most abundant ASVs assigned to each genus were consistent across all samples (Figure S6). In the unspiked sterile bentonite samples, contaminants constituted more than 96% of the total reads, reaching over 99% in four of six samples (Figure S7). Many spurious ASV were identified collectively representing only between 0.4% to 3.6% of the total relative abundance.

**FIGURE 5:**
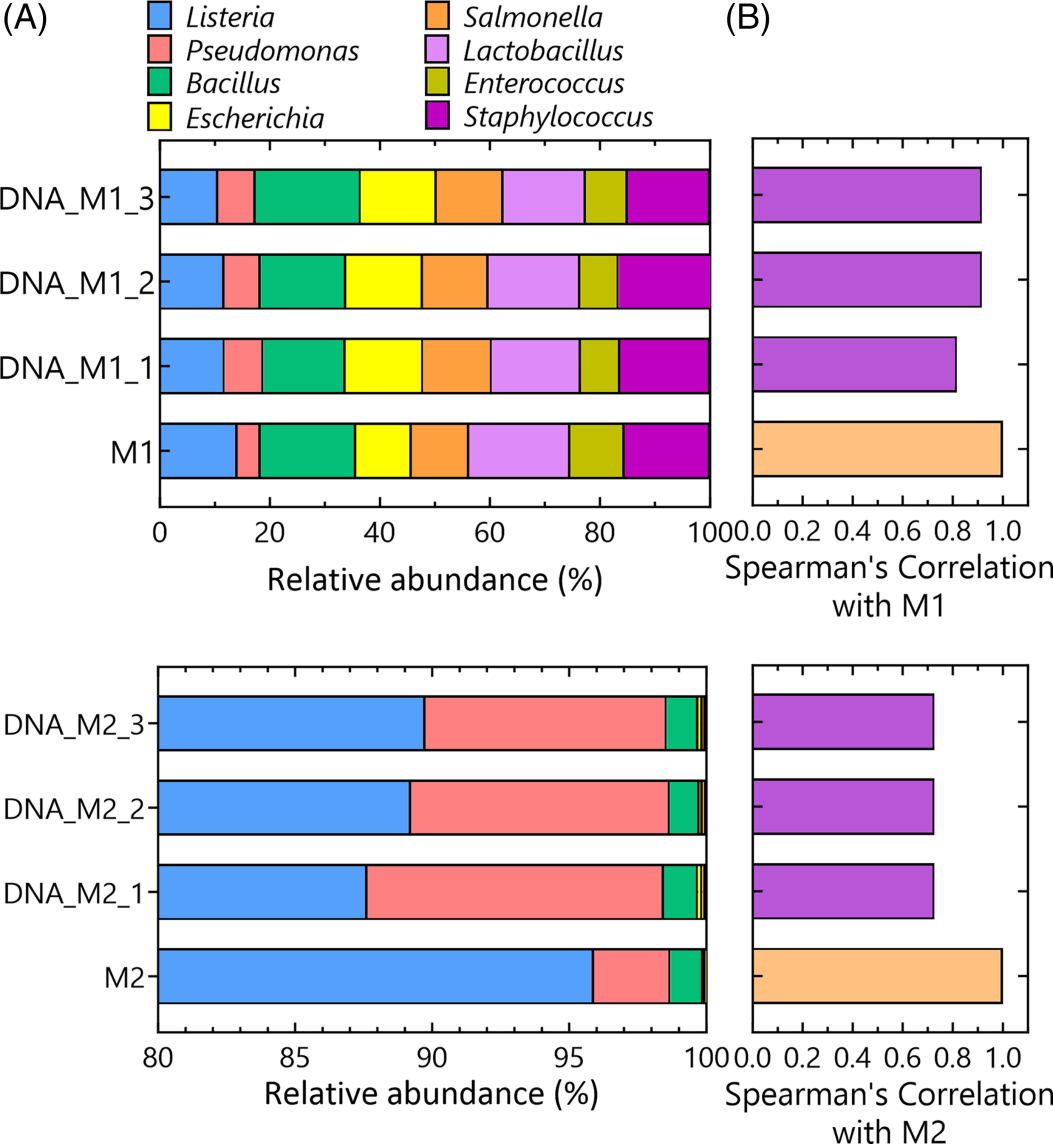
Bar plot showing the relative abundance of the genera present in the ZymoBIOMICS Microbial Community DNA Standard with a linear distribution of the species (DNA_M1) and a logarithmic distribution (DNA_M2) of the species. The theoretical distribution is represented as M1 and M2, respectively. Replicates are encoded as _replicate‐number (A). Spearman's correlation values (rho) for each replicate compared to the theoretical composition (B).

To assess potential PCR bias, we included controls with mock community standards composed of DNA instead of intact cells (Zymo Research Corporation, Irvine, USA). Sequencing results of these controls revealed minimal bias and demonstrated only minor variations across the different replicates (Figure 5). This was confirmed by high Spearman's correlation coefficients comparing the samples with the theoretical composition (Figure 5). The logarithmic distribution in Mock 2 allowed us to establish the detection limit. Our findings indicate that *Lactobacillus*, theoretically present with a relative abundance of 0.012% could be reliably identified in two out of three replicates. However, strains with lower distributions such as *Enterococcus* (0.001%) and *Staphylococcus* (0.0001%) remained undetectable (Figure 5). Spearman's correlation coefficients were slightly lower compared to those obtained with DNA of Mock 1, but the results still matched very well and were identical between replicates (*ρ* = 0.728).

**FIGURE 6:**
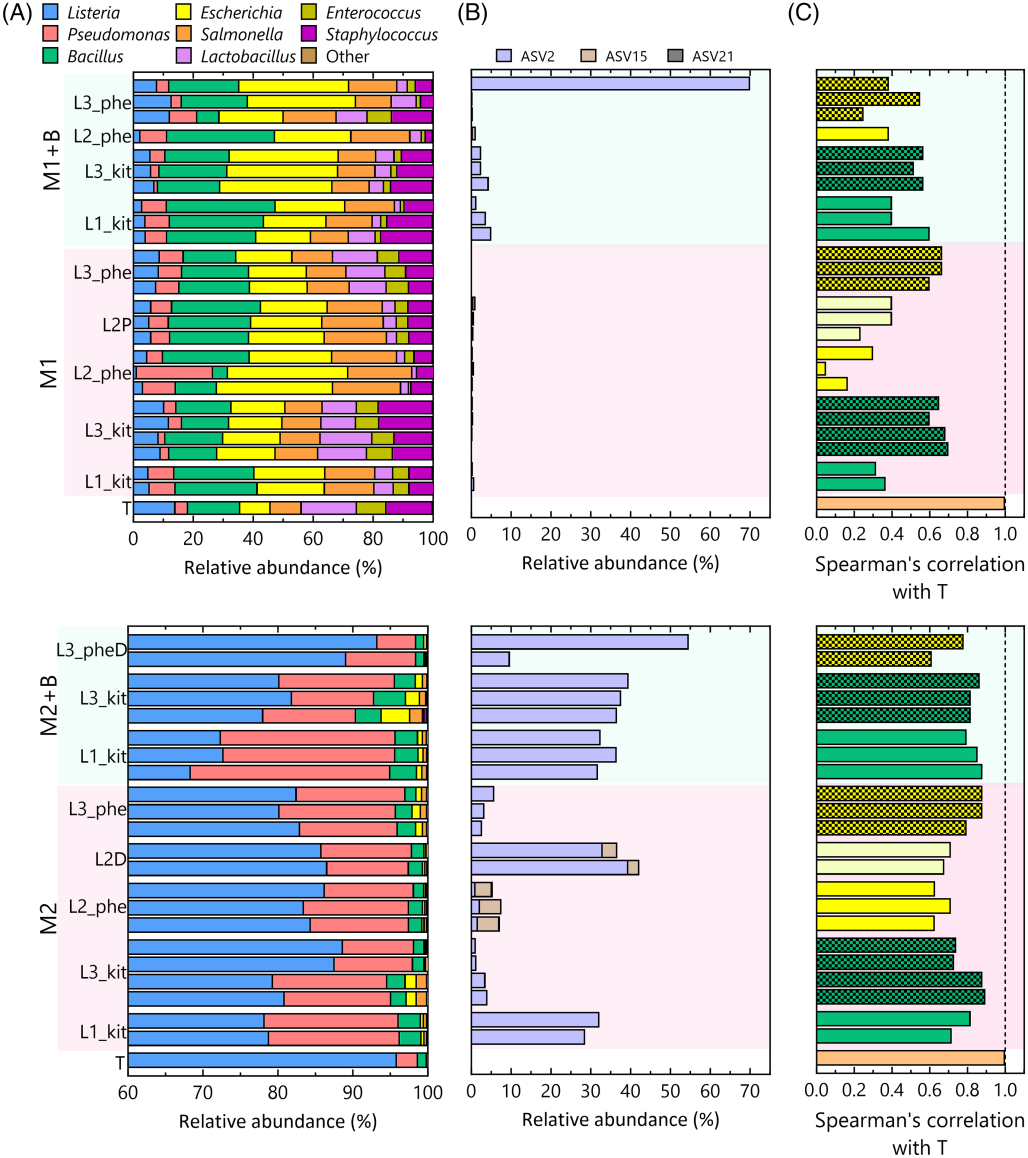
Bar plot showing the relative abundance of the genera present in samples spiked with Mock 1 or Mock 2 after removal of the contaminants (A). Relative abundance of ASVs identified as contaminants in samples with Mock 1 and Mock 2. Only ASVs with a relative abundance >0.1% are shown (B). Spearman's correlation coefficients compared to the theoretical distribution (T) (C). L1_kit and L3_kit represent results of the kit‐based extraction and are shown in green and dotted green; L2_phe and L3_phe are results from the phenol‐chloroform extraction are presented in yellow and dotted yellow. L2P represents samples after an additional phenol‐chloroform extraction according to the method of lab 3 and samples that are 40 times diluted as PCR template are designated with L2D. Samples diluted before a successful PCR reaction was obtained are indicated with L3_PheD. Samples without bentonite are shown in pink and samples with bentonite are coloured light green.

Samples spiked with Mock 1 demonstrated that both the kit‐based and phenol‐chloroform‐based DNA extraction methods were successful in capturing all species present in the mock community (Figure 6). Moreover, we observed minimal variation among replicates. However, higher variation was observed in the samples extracted with the phenol‐chloroform method (Figure 6). Notably, the variation in L2‐processed samples without bentonite decreased after applying an additional phenol‐chloroform extraction according to the method of lab 3 (Figure 6). The reason for the observed variation in samples processed by lab 3 in the presence of bentonite remains unclear. It is worth mentioning that the total DNA extracted from these samples also exhibited variation among the replicates and the relative abundance of ASVs identified as contaminants was 70% in one of the replicates (Figure 6). In all other samples spiked with Mock 1, the contribution of contaminants was very low. These observations were further supported by Spearman's correlation coefficients, which were compared to the theoretical composition (Figure 6). The highest correlations were observed for lab 3 regardless of the extraction method employed. Importantly, the presence of bentonite did not adversely affect the correlation with the theoretical composition.

The samples spiked with Mock 2 also showed minor variations among the different replicates. In addition, Spearman's correlation coefficients exhibited similarity across all conditions, with all samples achieving values above 0.6. However, the relative abundance of contaminant ASVs was generally much higher compared to the samples spiked with Mock 1, especially in the presence of bentonite. Important to note is that diluting the samples to mitigate the impact of PCR inhibitors could lead to a significant increase in the presence of contaminants (Figure 6).

**FIGURE 7:**
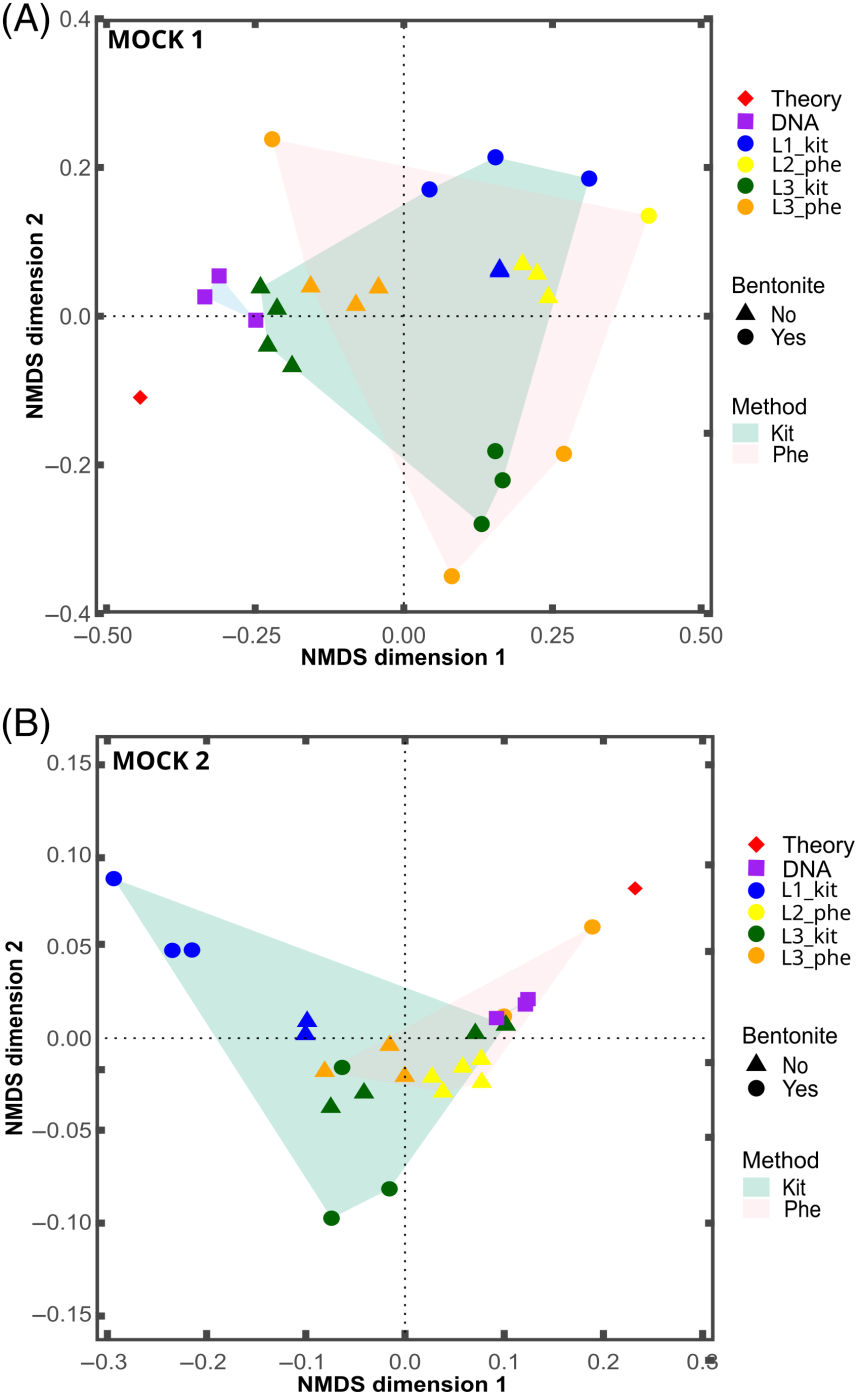
Non‐metric multidimensional scaling (NMDS) of amplicon sequence variant‐based bacterial community composition in samples spiked with (A) Mock 1 and (B) Mock 2, using Bray‐Curtis distances (stress = 0.13 and 0.02). Kit‐based approaches performed by lab 1 are shown in blue, while those performed by lab 3 are shown in green, with a green convex hull encompassing the group. Phenol‐chloroform‐based methods carried out by lab 2 are depicted in yellow, and those performed by lab 3 are shown in orange, grouped with a pink convex hull. Samples without bentonite are represented as triangles, while samples with bentonite are shown as circles. The DNA mock is depicted as purple squares, and the expected composition is represented by a red diamond.

As a rank‐based approach such as Spearman's correlation might not be suitable when only one or few strains are dominant, we also used non‐metric multidimensional scaling (NMDS) based on Bray Curtis distances to evaluate the diversity among the samples spiked with Mock 1 or Mock 2 between DNA extraction approaches (Figure 7). Most replicates are located closely together, except for bentonite spiked with Mock 1 processed by lab 3 with the phenol: chloroform‐based approach (L3_phe). Overall, this confirmed limited variability among replicates. Furthermore, samples without bentonite grouped better than samples with bentonite (Figure S8).

To statistically evaluate the impact of the DNA extraction method on the results, a PERMANOVA analysis was conducted on samples spiked with Mock 1 or Mock 2, including the DNA mock. Only samples without bentonite were included and PERMANOVA was performed for each mock community separately (Table S4). Prior to analysis, homogeneity of multivariate dispersions was assessed to ensure comparable variability among each group. The analysis revealed a significant effect of the DNA extraction method for both mock communities (*p* = 0.017 in case of Mock 1 and *p* = 0.003 in case of Mock 2). However, detailed pairwise comparisons between each group (each extraction method, DNA mock and theoretical composition) indicated no significant difference in microbial composition obtained by both methods or between each method and the theoretical expected composition. The only distinction observed was between the composition of the DNA mock and the samples extracted by the phenol‐chloroform‐based method spiked with Mock 1 and in samples spiked with Mock 2 (Table S4).

Additionally, to test the effect of bentonite on the performance of the two extraction methods, we first included all samples with and without bentonite (regardless of the DNA extraction method) and performed PERMANOVA on samples spiked with Mock 1 or with Mock 2. However, for Mock 1, a permutation test for homogeneity of multivariate dispersions was significant; thus no further PERMANOVA analysis could be conducted. Instead, we evaluated the effect of bentonite on the performance of extraction methods by comparing all samples with and without bentonite processed with the kit‐based extraction or with the phenol‐chloroform extraction (Table S5). This showed a significant effect of bentonite on each extraction method regardless of the Mock used (Table S5). However, detailed pairwise comparison of samples with and without bentonite and the theoretical Mock composition performed independently for each extraction method and Mock type were mostly non‐significant. The only significant difference between samples with and without bentonite was detected in case of Mock 1 samples processed using the kit‐based extraction (*p* = 0.025) and Mock 2 samples processed using the phenol‐chloroform extraction (*p* = 0.025).

## DISCUSSION

The goal of this study was to assess the representativeness of DNA extraction from clay by comparing two methods (Engel et al., 2019; Povedano‐Priego et al., 2021). To address this, we conducted an inter‐laboratory comparison of the two methods, with slight modifications, using Wyoming MX‐80 bentonite spiked with two mock communities. We compared the obtained DNA yield and purity (no requirement for additional purification steps), the presence of contaminants and the community profile, in the context of the clay and the two methods considered.

### DNA yields in the absence of bentonite

To detect possible differences between both extraction protocols and variability between the labs, we first focused on the extraction of DNA from both mock communities without bentonite. In these conditions, we observed no substantial variation in the DNA yield from samples processed with the phenol‐chloroform extraction. However, substantial differences were observed with the kit‐based extraction method (Figure 2). We therefore carefully evaluated the differences in the performed extraction protocol and performed additional experiments targeting the role of each difference. Our data indicate that increasing the volume of the elution buffer (both intermediate and final elution) in the procedure of lab 1 to the same level as that of lab 3 increases the DNA yields to the same level for both labs. The yields obtained in this study were at least three times higher than what was observed by a recent study comparing different DNA extraction methods on Mock 1 (Spreckels et al., 2023).

### DNA yields in the presence of bentonite

DNA extractions performed on the bentonite samples spiked with the mock community revealed that the presence of bentonite considerably hindered the efficiency of DNA extraction, except in samples processed by lab 3 using the phenol‐chloroform‐based extraction (Figure 2). Clay particles are known to tightly adsorb organic and inorganic phosphorous compounds, including DNA (Cai et al., 2007). Consequently, this significantly hinders the efficiency of DNA extraction (Frostegård et al., 1999). A 60%–80% adsorption of DNA of single strains was observed by bentonite and its minerals, which results in very low or unmeasurable amounts of DNA (Engel et al., 2019; Pietramellara et al., 2001; Stone et al., 2016). In addition, high variation in DNA yields was observed when Opalinus Clay was spiked with Mock 1 and extracted by seven different methods (Mijnendonckx et al., 2021). Variation in DNA yields between the methods was also observed in this study.

Overall, our results showed that in the presence of bentonite, the kit‐based procedure resulted in lower yields compared to the phenol‐chloroform extraction, particularly for Mock 2. Nevertheless, it consistently yielded highly pure DNA ready for downstream applications, as PCR amplification was successful in all cases. Similarly, a lower yield was obtained during kit‐based DNA extractions from bentonite (Engel, Coyotzi, et al., 2019) and in general when extracting DNA from environmental samples (Luna et al., 2006). Contrary, the kit‐based method resulted in the highest DNA yield when Opalinus Clay was spiked with Mock 1 (Mijnendonckx et al., 2021). Despite the higher DNA yields obtained with the phenol‐chloroform‐based method, several additional purification steps or dilutions were necessary before downstream applications were successful, especially in the case of lab 2 samples. We have not performed spectrophotometrical measurements (e.g., Nanodrop) to measure the purity of the samples as several samples had DNA concentrations that were too low to obtain reliable results. Instead, we scored the purity of the samples based on the possibility of amplifying them. In addition, it was visually evident which samples contained more impurities than others, depending on the extraction method used.

We observed marked differences in yields for both protocols among the labs. In case of kit‐based extractions, DNA yields were considerably lower for lab 1 than lab 3 for both Mock 1 and Mock 2 (Figure 2), which was consistent with the results of Mock‐only extractions. The lower yields may result from different elution volumes, as discussed above. The observed differences in purity and in obtained yields in case of the phenol‐chloroform extractions can potentially also be attributed to variations in the protocol employed. Lab 2 utilized a mixture of phenol:chloroform:isoamylalcohol followed by a washing step with pure chloroform, whereas lab 3 employed a mixture of chloroform and isoamylalcohol after the extraction with phenol:chloroform:isoamylalcohol. It has been shown previously that chloroform alone failed to produce a sharp interface between the aqueous and organic phase and a mixture of chloroform and isoamylalcohol is recommended (Lever et al., 2015). Additionally, lab 3 implemented additional washing steps with milli‐Q water after DNA precipitation. These two protocol modifications likely contributed to increased purity and improved efficiency in samples containing bentonite.

### Contaminants

One of the common issues of microbial community composition analysis in low‐biomass samples, such as bentonite, is the presence of contaminants (Salter et al., 2014). Failure to include appropriate controls and the resulting unrevealed presence of contaminant sequences can lead to substantial bias in result interpretation. A DNA template of 5 ng was used for PCR amplification of all samples, because it has been demonstrated to enhance amplification reproducibility compared to a lower amount of DNA template (Kennedy et al., 2014). However, some samples had DNA concentrations below the detection limit, necessitating the use of a lower amount of DNA template. This was especially true for the samples spiked with Mock 2 where the strains exhibited a logarithmic distribution and the total number of spiked cells was 10 times lower, which resulted in much lower DNA yields compared to the samples spiked with Mock 1. Lowering of the input DNA clearly led to an increased presence of contaminants, which was also observed when bentonite was spiked with low amounts of DNA from a pure strain (Engel, Coyotzi, et al., 2019). A similar effect was also observed after sample dilution necessary for successful amplification in case of some phenol‐chloroform extracted samples and thus starting from a lower amount of DNA template, which resulted in a significantly higher relative abundance of contaminant ASVs. To distinguish possible contaminants, we included NTCs for the PCR reaction and negative controls for the kit extraction. This approach facilitated the identification and exclusion of contaminants and showed that the observed differences between the samples spiked with Mock 1 and Mock 2 could largely be explained by the relative abundance of ASVs identified as contaminants. Sequencing results revealed that although 6 out of 12 unamended irradiated samples yielded positive PCR results, more than 96% of the total reads (and over 99% in four out of six samples) corresponded to contaminants (Figure S7). The remaining reads may have originated from dead microorganisms or spores, that persisted in the bentonite after irradiation but represented an insignificant fraction. Our findings validate that irradiation can be considered as an effective method for obtaining sterile bentonite samples, which can serve as negative controls in microbiome studies involving bentonite. Moreover, this study demonstrates the importance of vigilance regarding the presence of contaminant sequences in samples from low‐biomass environments where the obtained DNA concentration is often low.

### Microbial community composition

Contrary to the observed variation in the total amount of DNA obtained from the samples, after the removal of the contaminant ASVs, the microbial community composition showed relatively limited variation. In the absence of bentonite, the two methods evidenced a consistent microbial community corresponding to the expected composition. This suggests that both methods are suitable for DNA extraction from a mixture of microbial strains with different characteristics. However, the presence of bentonite had a significant effect on the microbial composition of samples for both methods. This result might be due to the overall lower DNA yields from bentonite samples and highlights the possible negative impact of bentonite on extraction efficiency. Nevertheless, no differences compared to the theoretical composition were observed in bentonite samples extracted by both extraction methods and Spearman's correlation coefficients were similar in most samples independent of the presence of bentonite. In fact, Spearman's correlation coefficients were higher and more consistent among the samples spiked with Mock 2, regardless of the DNA extraction method employed. In Mock 2, one ASV is highly dominant so our results indicate that both methods are highly robust in conditions where only one (or a few) species is/are dominant. More variation was observed in samples spiked with Mock 1, where the species were more equally abundant. However, variability was more pronounced among different laboratories than it was dependent on the presence of bentonite. Consequently, both methods seem to provide a reliable representation of the actual microbial composition in the Mock samples spiked in the bentonite, despite the described differences.

## CONCLUSION

We conducted an inter‐laboratory comparison of two DNA extraction methods on MX‐80 bentonite. Our results, along with previously published evidence, clearly demonstrate that even minor changes in extraction protocols can significantly affect both the efficiency and purity of the extracted DNA. Differences in DNA yield based on the choice of extraction method can be important, especially since bentonite is typically a low‐biomass environment. Importantly, our findings indicate that the choice between the two methods is not critical, as each has advantages. However, retaining consistency in the chosen method is essential, as comparing results becomes challenging, particularly in the presence of bentonite.

In general, the kit‐based method with an intermediate elution in 2.3 mL and further purification with the Genomic DNA Clean & Concentrator™ Kit and a final elution in 125 μL is the preferred procedure, as it results in highly pure DNA and is the least time‐consuming. However, pooling a large number of samples might be necessary to obtain sufficient DNA using this method if very low biomass is present. In such cases, the phenol‐chloroform‐based method appears to be the optimal choice as it yields a higher amount of DNA. However, this method is more time‐consuming and may be more susceptible to impurities in the final DNA sample and technical variations. It is recommended to wash with a mixture of chloroform and isoamyl alcohol and to implement additional washing steps after DNA precipitation to obtain amplifiable DNA.

Lastly, our findings emphasize the importance of including appropriate controls when working with challenging samples, particularly those with low biomass.

## AUTHOR CONTRIBUTIONS

**Kristel Mijnendonckx:** Conceptualization; methodology; funding acquisition; visualization; writing – review and editing; writing – original draft; formal analysis; data curation; project administration. **Carla Smolders:** Investigation; methodology. **Deepa Bartak:** Investigation; methodology; writing – review and editing; writing – original draft. **Trung Le Duc:** Methodology; investigation. **Mar Morales‐Hidalgo:** Investigation; methodology; writing – review and editing. **Cristina Povedano‐Priego:** Investigation; methodology; writing – original draft; writing – review and editing. **Fadwa Jroundi:** Methodology; supervision; writing – review and editing. **Mohamed L. Merroun:** Funding acquisition; writing – review and editing; supervision; resources. **Natalie Leys:** Writing – review and editing; funding acquisition; resources; supervision. **Katerina Cerna:** Conceptualization; investigation; methodology; writing – review and editing; writing – original draft; supervision; resources; funding acquisition.

## CONFLICT OF INTEREST STATEMENT

The authors declare no conflicts of interest.

## Supporting information


**Data S1.** Supporting information.

## Data Availability

The datasets generated and analyzed during this study are available in the NCBI Sequence Read Archive (SRA) repository (PRJNA1054184): https://www.ncbi.nlm.nih.gov/bioproject/PRJNA1054184.
