# Supplementary material for: Comparing the effectiveness of different DNA extraction methods in MX‐80 bentonite
Source: Environ Microbiol Rep. 2024 Nov 24;16(6):e70047. doi: 10.1111/1758-2229.70047 (PMC11586505; doi:10.1111/1758-2229.70047)
Supplement: Supplementary file 1 — Data S1. Supporting information. [file EMI4-16-e70047-s001.docx]

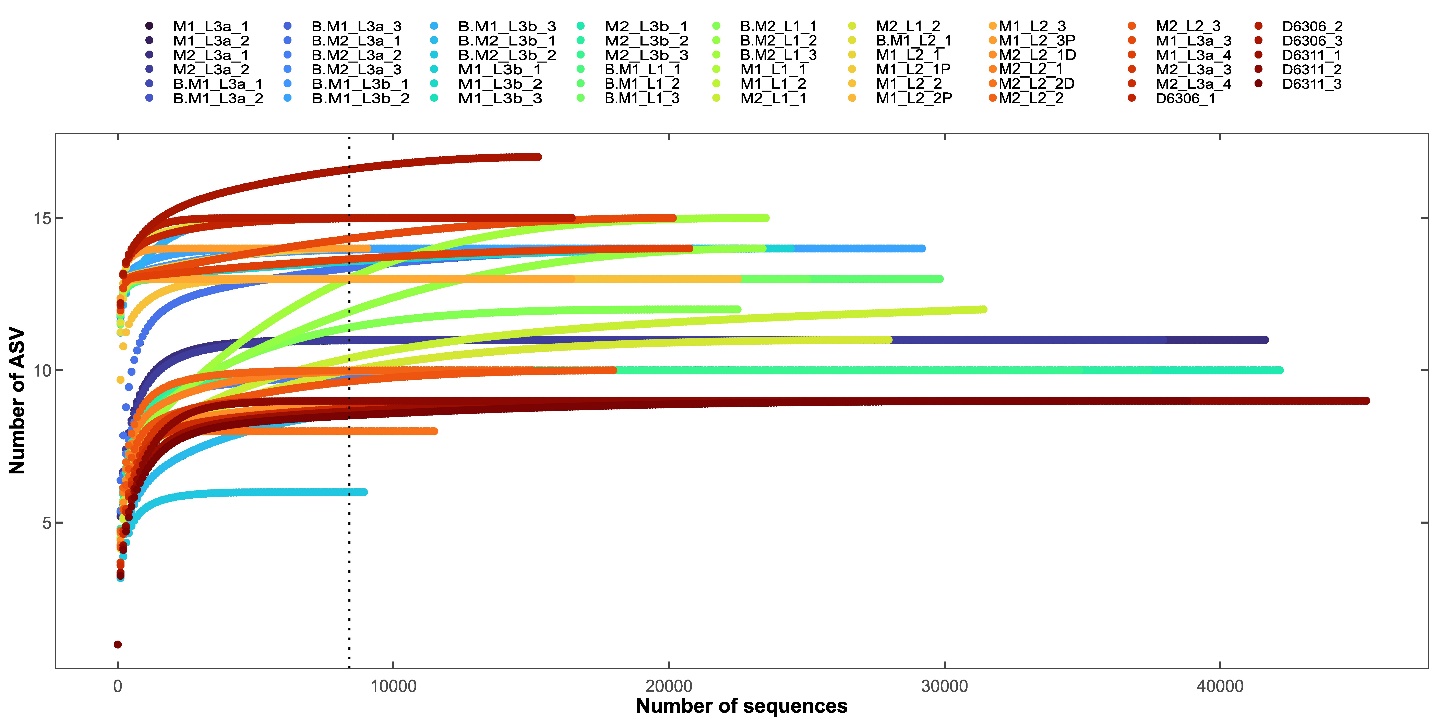


Supplementary Figure 1: Rarefaction curves showing the number of ASVs in the function of the number of reads.


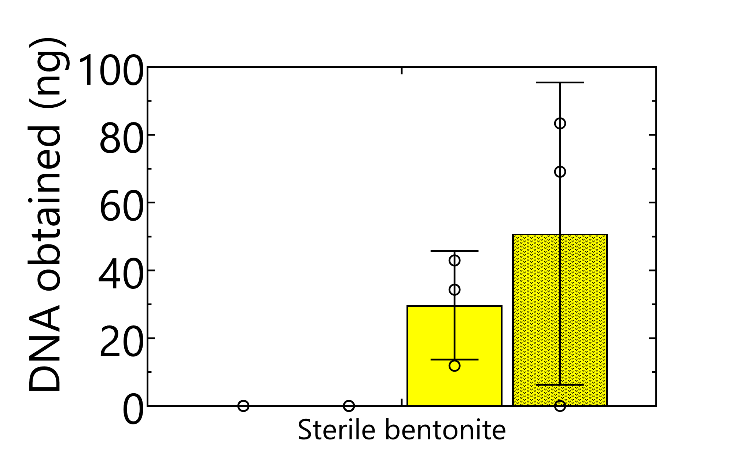


Supplementary Figure 2: Total amount of DNA obtained from irradiated bentonite samples with a phenol-chloroform-based extraction method performed by lab 2 (yellow) and lab 3 (dotted yellow). Data represent the average and standard deviation of three replicates.


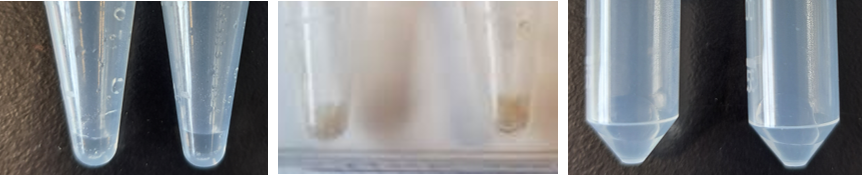


Supplementary Figure 3: Result of the solution after the DNA extraction for two replicates of lab 1 (left), lab 2 (middle) and lab 3 (right).


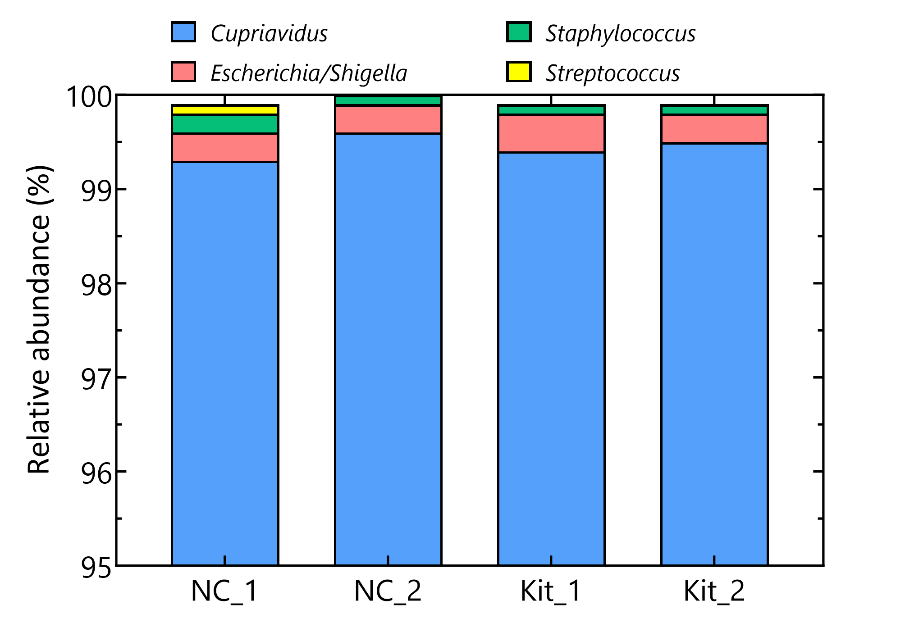


Supplementary Figure 4: Bar plot showing the relative abundance of the three most dominant genera present in the NTC samples of the PCR (NC) and the kit controls (Kit). Different replicates are encoded as _replicate.


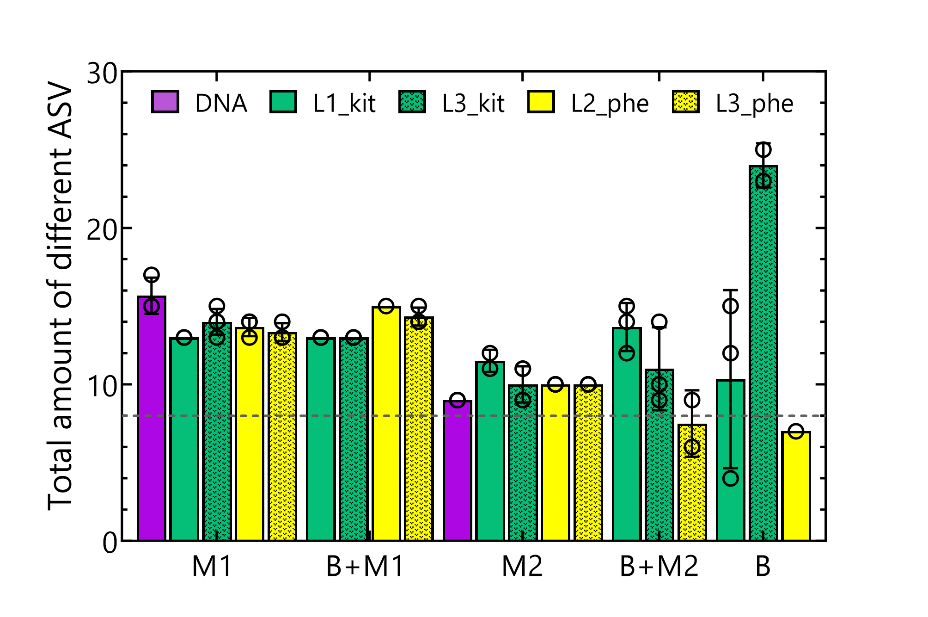


Supplementary Figure 5: Total amount of different ASV identified in the samples spiked with Mock 1 in the absence of bentonite (M1) or in the presence of bentonite (B+M1); in the samples spiked with Mock 2 in the absence of bentonite (M2) or in the presence of bentonite (B+M2); in unspiked sterile bentonite (B). The expected number of ASVs present in the mock is shown by a dotted line.


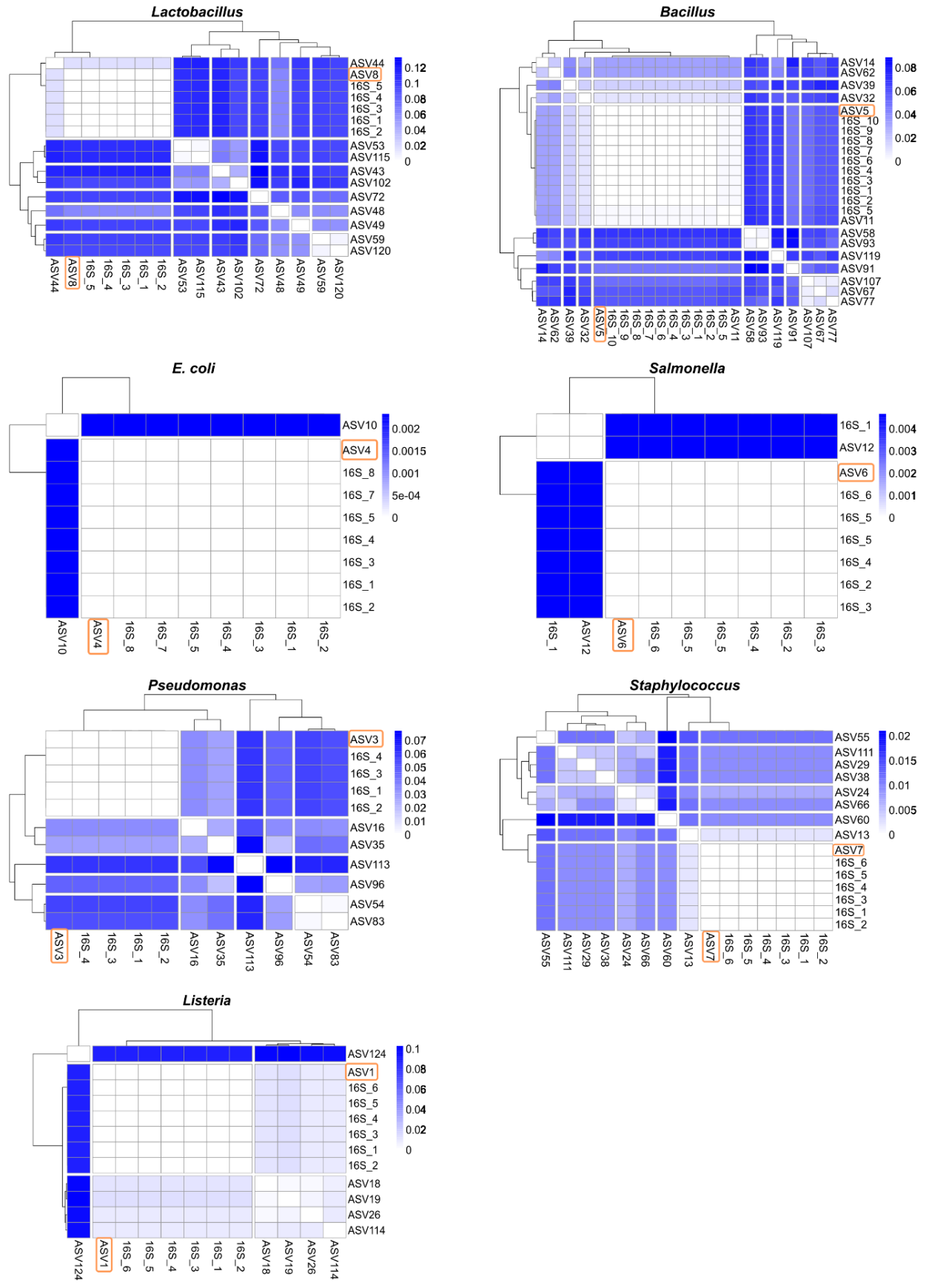


Supplementary Figure 6: Distance matrices based on multiple alignments of the 16S rRNA amplicons of the strains present in the ZymoBIOMICS mock (16S_x) compared to the ASV present in the samples (ASVx). The most dominant ASV is indicated by an orange square.


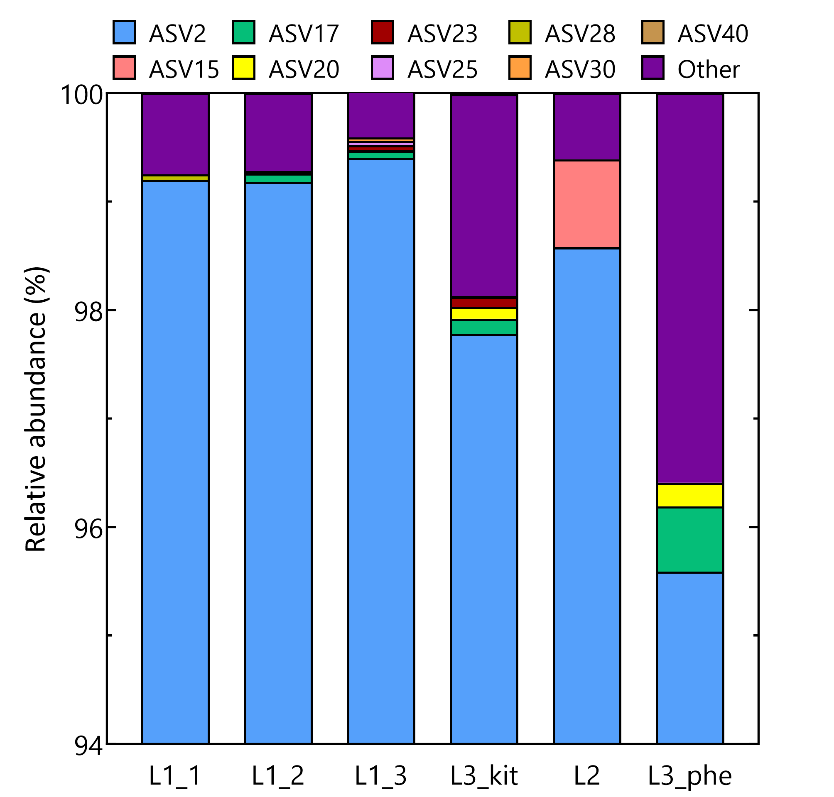


Supplementary Figure 7: Bar plot showing the relative abundance of the ASV determined as being contaminants in the unspiked sterile bentonite samples that showed a positive PCR amplification. One contaminant ASV21 is not shown as it was not present in these samples.


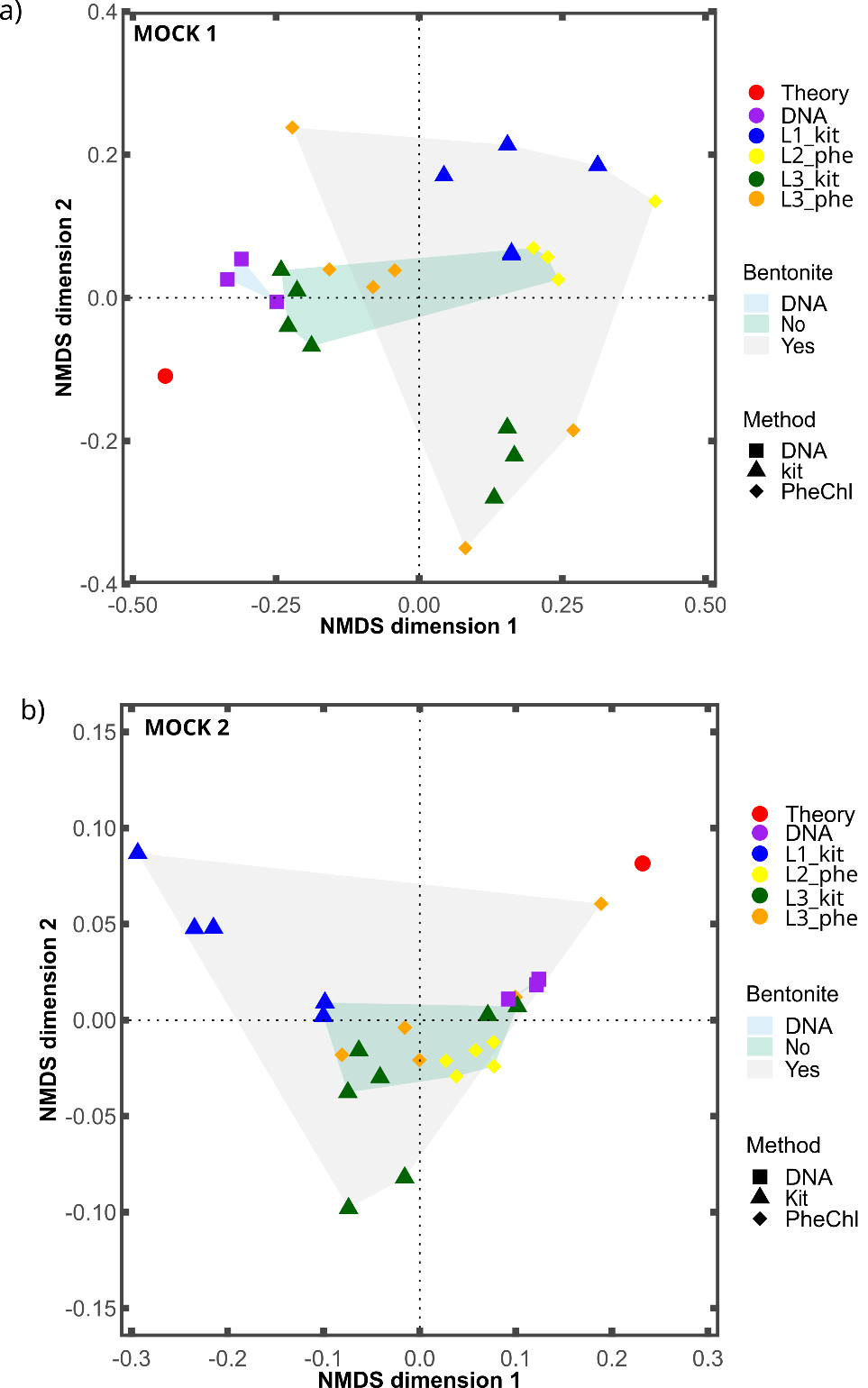


Supplementary Figure 8: Nonmetric Multidimensional Scaling (NMDS) of ASV-based bacterial community composition in samples spiked with a) Mock 1 and b) Mock 2, using Bray-Curtis distances (stress = 0.13 and 0.02). Kit-based approaches performed by lab 1 are shown in blue triangles, while those performed by lab 3 are shown as green triangles. Phenol-chloroform-based methods carried out by lab 2 are depicted as yellow diamonds, and those performed by lab 3 are shown as orange diamonds. Samples without bentonite are grouped with a green convex hull, while samples with bentonite grouped as gray convex hull. The DNA mock is depicted as purple squares and grouped with a pink convex hull, and the expected composition is represented by a red circle.

Supplementary Table 1: Composition of microbial communities in the ZymoBIOMICS Microbial Community Standards used for this study.

| **Species** | **NRRL^a^ accession number** | **Gram stain** | **Genome size (Mb)** | **16S/18S copy number** |
| --- | --- | --- | --- | --- |
| *Pseudomonas aeruginosa* | B-3509 | - | 6.79 | 4 |
| *Escherichia coli* | B-1109 | - | 4.88 | 7 |
| *Salmonella enterica* | B-4212 | - | 4.76 | 7 |
| *Lactobacillus fermentum* | B-1840 | + | 1.91 | 5 |
| *Enterococcus faecalis* | B-537 | + | 2.85 | 4 |
| *Staphylococcus aureus* | B-41012 | + | 2.73 | 6 |
| *Listeria monocytogenes* | B-33116 | + | 2.99 | 6 |
| *Bacillus subtilis* | B-354 | + | 4.05 | 10 |
| *Saccharomyces cerevisiae* | Y-567 | Yeast | 12.1 | 109^b^ |
| *Cryptococcus neoformans* | Y-2534 | Yeast | 18.9 | 60^b^ |
| ^a^Agricultural Research Service Culture Collection  ^b^Estimated based on read depth information from mapping shotgun sequencing data | | | | |

Supplementary Table 2: Overview of the samples sent for 16S rRNA amplicon sequencing. Samples spiked with a mock community but without successful PCR reaction after further extraction, purification or dilution (-) were not sent for sequencing. DNA yields were estimated directly after the DNA extraction and prior to further dilution/purification/re-extraction steps needed to obtain positive PCR in some of the samples. >DL: under the detection limit, NA: not analyzed.

| **Lab** | **Method** | **Bentonite** | **Mock** | **Replicate** | **DNA yield (ng)** | **PCR successful** |
| --- | --- | --- | --- | --- | --- | --- |
| 1 | Kit | No | M1 | 1 | 47.6 | + |
|  |  |  |  | 2 | 104.5 | + |
|  |  |  |  | 3 | 80.5 | NA |
|  |  |  | M2 | 1 | 1.1 | + |
|  |  |  |  | 2 | 1.9 | + |
|  |  |  |  | 3 | 4.5 | NA |
|  |  | Yes | M1 | 1 | 4.5 | + |
|  |  |  |  | 2 | 1.8 | + |
|  |  |  |  | 3 | 1.2 | + |
|  |  |  | M2 | 1 | <DL | + |
|  |  |  |  | 2 | <DL | + |
|  |  |  |  | 3 | <DL | + |
|  |  |  | No | 1 | <DL | + |
|  |  |  |  | 2 | <DL | + |
|  |  |  |  | 3 | <DL | + |
|  |  | No | No (NC) | 1 | <DL | + |
|  |  |  |  | 2 | <DL | + |
| 2 | PheChl | No | M1 | 1 | 1008.8 | + |
|  |  |  |  |  |  | Additional PheChl extraction + Amicon |
|  |  |  |  | 2 | 830.3 | + |
|  |  |  |  |  |  | Additional PheChl extraction + Amicon |
|  |  |  |  | 3 | 664 | + |
|  |  |  |  |  |  | Additional PheChl extraction + Amicon |
|  |  |  | M2 | 1 | 49.0 | Drop dialysis |
|  |  |  |  |  |  | Drop dialysis & 40 times dilution |
|  |  |  |  | 2 | 46.9 | Drop dialysis |
|  |  |  |  |  |  | Drop dialysis & 40 times dilution |
|  |  |  |  | 3 | 44.2 | Additional PheChl extraction + Amicon |
|  |  | Yes | M1 | 1 | 202.3 | Additional PheChl extraction + Amicon |
|  |  |  |  | 2 | 42.0 | - |
|  |  |  |  | 3 | 36.6 | - |
|  |  |  | M2 | 1 | 27.8 | - |
|  |  |  |  | 2 | 27.5 | - |
|  |  |  |  | 3 | 27.5 | - |
|  |  |  | No | 1 | 34.3 | Additional PheChl extraction + Amicon |
|  |  |  |  | 2 | 11.9 | - |
|  |  |  |  | 3 | 42.9 | - |
| 3 | Kit | No | M1 | 1 | 765.1 | + |
|  |  |  |  | 2 | 1281.5 | + |
|  |  |  |  | 3 | 647.1 | + |
|  |  |  |  | 4 | 573.7 | + |
|  |  |  | M2 | 1 | 52.3 | + |
|  |  |  |  | 2 | 112.0 | + |
|  |  |  |  | 3 | 149.6 | + |
|  |  |  |  | 4 | 140.4 | + |
|  |  | Yes | M1 | 1 | 194.5 | + |
|  |  |  |  | 2 | 322.5 | + |
|  |  |  |  | 3 | 147.0 | + |
|  |  |  | M2 | 1 | 22.7 | + |
|  |  |  |  | 2 | 9.7 | + |
|  |  |  |  | 3 | 9.4 | + |
|  |  |  | No | 1 | <DL | + |
|  |  |  |  | 2 | <DL | + |
|  |  |  |  | 3 | <DL | - |
|  | PheChl | No | M1 | 1 | 1231.6 | + |
|  |  |  |  | 2 | 1158.7 | + |
|  |  |  |  | 3 | 1073.4 | + |
|  |  |  | M2 | 1 | 42.2 | + |
|  |  |  |  | 2 | 114.5 | + |
|  |  |  |  | 3 | 30.7 | + |
|  |  | Yes | M1 | 1 | 2066.2 | + |
|  |  |  |  | 2 | 2399.6 | + |
|  |  |  |  | 3 | 707.7 | + |
|  |  |  | M2 | 1 | 121.3 | Drop dialysis + 40 times dilution |
|  |  |  |  | 2 | 123.8 | Drop dialysis + 40 times dilution |
|  |  |  |  | 3 | 189.8 | - |
|  |  |  | No | 1 | 83.4 | - |
|  |  |  |  | 2 | 69.1 | - |
|  |  |  |  | 3 | <DL | - |

Supplementary Table 3: Overview of ASVs identified as contaminants

| **ASV** | **Phylum** | **Class** | **Order** | **Family** | **Genus** |
| --- | --- | --- | --- | --- | --- |
| ASV2 | *Proteobacteria* | *Gammaproteobacteria* | *Betaproteobacteriales* | *Burkholderiaceae* | *Cupriavidus* |
| ASV15 | *Proteobacteria* | *Gammaproteobacteria* | *Pseudomonadales* | *Pseudomonadaceae* | *Pseudomonas* |
| ASV17 | *Firmicutes* | *Bacilli* | *Bacillales* | *Staphylococcaceae* | *Staphylococcus* |
| ASV20 | *Firmicutes* | *Bacilli* | *Lactobacillales* | *Streptococcaceae* | *Streptococcus* |
| ASV21 | *Firmicutes* | *Bacilli* | *Bacillales* | *Listeriaceae* | *Listeria* |
| ASV23 | *Firmicutes* | *Bacilli* | *Bacillales* | *Family_XI* | *Gemella* |
| ASV25 | *Firmicutes* | *Bacilli* | *Bacillales* | *Bacillaceae* | *Aeribacillus* |
| ASV28 | *Proteobacteria* | *Gammaproteobacteria* | *Pseudomonadales* | *Moraxellaceae* | *Enhydrobacter* |
| ASV30 | *Proteobacteria* | *Gammaproteobacteria* | *Pseudomonadales* | *Pseudomonadaceae* | *Pseudomonas* |
| ASV40 | *Firmicutes* | *Bacilli* | *Bacillales* | *Staphylococcaceae* | *Staphylococcus* |

Supplementary Table 4: Overview of the different PERMANOVA and pairwise comparisons performed to estimate the effect of DNA extraction methods on the microbial community composition. Only samples without bentonite were included. For the pairwise comparisons, only significant adjusted p-values (<0.05) are shown.

| **Effect of the DNA extraction method** | | | | | |
| --- | --- | --- | --- | --- | --- |
| **PERMANOVA test on Mock 1 (p=0.017)** | | | | | |
| **Pairwise comparisons** | Kit | PheChl | DNA mock | Theory |  |
| Kit |  |  |  |  |  |
| PheChl |  |  | 0.033 |  |  |
| DNA mock |  | 0.033 |  |  |  |
| Theory |  |  |  |  |  |
| **PERMANOVA test on Mock 2 (p=0.003)** | | | | |  |
| **Pairwise comparisons** | Kit | PheChl | DNA mock | Theory |  |
| Kit |  |  |  |  |  |
| PheChl |  |  | 0.013 |  |  |
| DNA mock |  | 0.013 |  |  |  |
| Theory |  |  |  |  |  |

Supplementary Table 5: Overview of the different permanova and pairwise comparisons performed to estimate the effect of the bentonite on both DNA extraction methods. For the pairwise comparisons, only significant adjusted p-values (<0.05) are shown.

| **Mock 1** | | | | | |
| --- | --- | --- | --- | --- | --- |
| **Effect of the bentonite for the kit extraction method (p=0.004)** | | | | |  |
| **Pairwise comparisons** | Without bentonite | With bentonite | Theory |  |  |
| Without bentonite |  | 0.025 |  |  |  |
| With bentonite | 0.025 |  |  |  |  |
| Theory |  |  |  |  |  |
|  |  |  |  |  |  |
| **Effect of the bentonite for the PheChl extraction method (p=0.042)** | | | | |  |
| **Pairwise comparisons** | Without bentonite | With bentonite | Theory |  |  |
| Without bentonite |  |  |  |  |  |
| With bentonite |  |  |  |  |  |
| Theory |  |  |  |  |  |
| **Mock 2** | | | | |  |
| **Effect of the bentonite for the kit extraction method (p=0.007)** | | | | |  |
| **Pairwise comparisons** | Without bentonite | With bentonite | Theory |  |  |
| Without bentonite |  |  |  |  |  |
| With bentonite |  |  |  |  |  |
| Theory |  |  |  |  |  |
|  |  |  |  |  |  |
| **Effect of the bentonite for the PheChl extraction method (p=0.006)** | | | | |  |
| **Pairwise comparisons** | Without bentonite | With bentonite | Theory |  |  |
| Without bentonite |  | 0.025 |  |  |  |
| With bentonite | 0.025 |  |  |  |  |
| Theory |  |  |  |  |  |
